# For the Practice of Medicine in the United States

**Published:** 1894-04

**Authors:** 


### LEGAL REQUIREMENTS FOR THE PRACTICE OF MEDICINE IN THE UNITED STATES.

So many changes have been made in the legislation regulating the practice of medicine in this country during the past three years that the Illinois State Board of Health will include in its forthcoming Report on Medical Education the text of all laws on this subject in force at the beginning of the present year in the several States and Territories of the United States, and in the provinces of the Dominion of Canada.

Of the six New England States, Maine, Massachusetts, New Hampshire and Rhode Island have no legal requirements for the practice of medicine. Connecticut has adopted medical a practice act which went into effect October 1, 1863, and in Vermont the law requires the registry of a diploma indorsed by a Board of Medical Censors, or a certificate of satisfactory examination by one of the boards.

Exclusive of the four States first named, the other States and Territories may be roughly grouped in the following three classes:

In Alabama, Arkansas, Florida, Maryland, Minnesota, Mississippi, New Jersey, New York (Act of May 9, 1893), North Carolina, North Dakota, Pennsylvania (after March 1, 1894), South Dakota, Texas, Utah, Virginia and Washington, the diploma confers no right to practice and has no legal value, except in some cases to give its possessor standing before an examining board. The right to practice in each of these sixteen States is determined by individual examination before boards of examiners created by law.

In California, Colorado, Connecticut (since October, 1893), Delaware, Illinois, Iowa, Kentucky, Louisiana, Missouri, Montana, Nebraska, New Mexico, Oklahoma, Oregon, Tennessee, Vermont and West Virginia, the diploma is subject to supervision of some designated body vested by law with authority to determine its validity as evidence of its possessor's qualifications for the practice of medicine. Failing the possession of such a recognized diploma, the right to practice may be acquired by passing a satisfactory examination.

In Arizona, Georgia, Idaho, Indiana, Kansas, Michigan, Nevada, Ohio, South Carolina (since the repeal of the Act of 1888), Wisconsin and Wyoming, the presentation of any kind of a diploma—provided only that it be from a "chartered" medical institution—is the sufficient warrant in law for county clerks, clerks of courts, registrars of deeds and similarly qualified judges of medical fitness to admit to practice.

Following is a *résumé* of the legal requirements for practice in each State and Territory in force January 1, 1894:

Alabama.—A certificate of successful examination by the State (or a county) Board of Medical Examiners. Diplomas confer no right to practice.

Arizona.—Registry with a county recorder of an unrevoked, uncancelled "diploma regularly issued by a medical college properly and lawfully organized under the laws of the State wherein said college shall be located."

Arkansas.—A certificate of successful examination by the State (or a county) Board of Medical Examiners. Diplomas confer no right to practice.

California.—A certificate issued on the diploma of a college in good standing, or upon a successful examination by one of the State Boards of Medical Examiners—regular, homeopathic or eclectic.

Colorado.—Similar to California, except that there is but one State Board of Examiners.

Connecticut.—A certificate of registration of the diploma of a college "recognized as reputable by one of the chartered medical societies of the State," regular, homeopathic, eclectic; or a certificate of satisfactory examination by a committee appointed for the purpose by the State Board of Health.

Delaware.—A certificate based upon the registration of a diploma from "a respectable medical college," or upon "a full and impartial examination by the State Board of Medical Examiners."

District of Columbia.—Nominally the indorsement of a diploma or an examination by a committee of the District Medical Society; practically no requirement.

Florida.—A certificate of satisfactory examination by the State

(or a district) Board of Medical Examiners. Diplomas confer no right to practice.

Georgia.—The registration of a diploma from any "incorporated medical college, medical school or university." The clerks of the Superior Courts are the sole judges of the diploma as evidence of fitness for medical practice.

Idaho.—The record of a diplima at a county seat.

Illinois.—A certificate issued by the State Board of Health upon the diploma of a legally chartered medical institution in good standing, as determined by the board, or upon a satisfactory examination by the board.

Indiana.—The registration in a county clerk's office of a diploma "from some reputable medical college."

Indian Territory.—*a.* Cherokee Nation: An examination by the Board of Medical Examiners; *b.* Choctaw Nation: A certificate by the Board of Medical Examiners; *c.* Creek Nation: Payment of $25 annually as a license fee.

Iowa.—Similar to Illinois.

Kansas.—The registry of a diploma from some "respectable school of medicine," or of a certificate of qualification from some State or county medical society.

Kentucky.—A certificate from the State Board of Health issued upon the "diploma of a reputable and legally chartered medical college."

Louisiana.—The record of a diploma from "any medical institution of credit and respectability" after indorsement by the State Board of Health.

Maine.—No legal requirement. In 1887 an act to regulate the practice of medicine was passed by the legislature but was vetoed by the governor.

Maryland.—A certificate issued by the State Board of Medical Examiners. Diplomas confer no right to practice.

Massachusetts.—No legal requirement.

Minnesota.—Similar to Maryland.

Mississippi.—Similar to Maryland, except that the examination is made and the certificate issued by the State Board of Health.

Missouri.—Similar to Illinois.

Montana.—Ten years of practice; a certificate upon the diploma of a college "in good standing," or upon an examination by the State Board of Medical Examiners.

Nebraska.—A certificate issued by the State Board of Health upon the diploma of a "legally chartered medical school or college in good standing," as defined in section 8 of the Act of July, 1891.

Nevada.—The record of a diploma from "some regularly chartered medical school."

New Hampshire.—No legal requirement.

New Jersey.—A license issued upon a successful examination by the State board of Medical Examiners. Diplomas confer no right to practice.

New Mexico—A certificate upon the diploma of a legally chartered medical institution in good standing, or an examination by Territorial Board of Medical Examiners.

New York.—A license issued upon a successful examination by one of the State Boards of Medical Examiners—regular, homeopathic, eclectic. Diplomas confer no right to practice.

North Carolina.—A license issued upon a successful examination by the State Board of Medical Examiners. Diplomas confer no right to practice.

North Dakota.—Similar to North Carolina.

Ohio.—The diploma of a respectable school of medicine, or a certificate of qualification from a State or county medical society.

Oklahoma.—A license issued by a Superintendent of Public Health upon a medical diploma or after examination.

Oregon.—A certificate on the diploma of a college "in good standing," or after examination by the State Board of Medical Examiners.

Pennsylvania.—A license issued after examination before one of the State Boards of Medical Examiners; Act of May 18, 1893; takes effect March 1, 1894. Diplomas will thereafter confer no right to practice.

Rhode Island.—No legal requirement.

South Carolina.—A certificate of verification of the diploma of a reputable medical college. An Act of December 24, 1890, abolished the State Board of Medical Examiners created by the Act of 1888 and under which the diploma conferred no right to practice.

South Dakota.—A license issued by the State Board of Health after examination. Diplomas confer no right to practice.

Tennessee.—A license on the diploma of a college "in good standing," or after examination by the State Board of Medical Examiners.

Texas.—A license issued after examination by a District Board of Medical Examiners. Diplomas confer no right to practice.

Utah.—A license issued by the Territorial Board of Medical Examiners after examination. Diplomas confer no right to practice.

Vermont.—The registry of a diploma indorsed by one of the Boards of Medical Censors, or a certificate of examination by one of the Boards.

Virginia.—A license issued after examination by the State Board of Medical Examiners. Diplomas confer no right to practice.

Washington.—Similar to Virginia.

West Virginia.—A license on the diploma of a reputable college, or after examination by the State Board of Health.

Wisconsin.—The indorsement of a medical diploma by the censors of either of the State or county medical societies.

Wyoming.—The record of a diploma with a registrar of deeds. —*J. A. M. A.*

---

### ICHTHYOL IN ERYSIPELAS.

Thomas (Liverpool *Medico-Chirurgical Journal,* July, 1893) refers to the treatment of erysipelas by ichthyol, and mentions four cases so treated, three of which were complicated by large surgical wounds. The onset of the disease was sudden, and the temperature high. As the result of the treatment, the disease was cured on the fifth day. In only one case was there sleeplessness. None required stimulants, and all experienced great relief from pain after each application of the remedy. Success in this treatment depends upon a very thorough rubbing of a strong ointment of ichthyol with vaseline or lanolin into the red area and into the adjoining healthy skin, covering the parts with a sheet of lint, or the ordinary surgical dressing.—*Therapeutic Gazette.*

